# Improving the Cardiovascular Outcomes of Obstructive Sleep Apnea: Towards More Precise Hypoxia-Based Models of Disease Severity

**DOI:** 10.1007/s40675-024-00315-7

**Published:** 2025-01-03

**Authors:** Nida T. Qayyum, Andrew T. Cole, Rami N. Khayat, Anna Grosberg

**Affiliations:** 1 Department of Chemical and Biomolecular Engineering, University of California, Irvine, Irvine, CA USA; 2 Cardiovascular Innovation and Research Center (CIRC), UCI Edwards Lifesciences Foundation, University of California, Irvine, Irvine, CA USA; 3 Department of Biomedical Engineering, University of California, Irvine, Irvine, CA 92698 USA; 4 The UCI Sleep Disorders Center, University of California, Irvine, Irvine, CA 92698 USA; 5 Center for Complex Biological Systems, University of California, Irvine, Irvine, CA USA; 6 NSF-Simons Center for Multiscale Cell Fate Research, University of California, Irvine, Irvine, CA USA; 7 Sue and Bill Gross Stem Cell Research, University of California, Irvine, Irvine, CA USA

**Keywords:** Obstructive sleep apnea, Apnea hypopnea index, Hypoxia burden, Mathematical modeling, Tissue oxygenation

## Abstract

**Purpose of Review:**

Obstructive sleep apnea (OSA) affects at least 1 billion people worldwide, and its increasing prevalence is alarming considering an association to comorbidities such as cardiovascular disease (CVD) and to demonstrated health disparities. This raises concerns regarding the current diagnostic standards, which are also impacted by disparities. The current review was aimed at identifying limitations in the apnea-hypopnea index (AHI), the primary clinical indicator of OSA severity, and analyzing recent alternatives. In addition, the association between OSA and CVD was discussed, and, considering the role of intermittent hypoxia, solutions were proposed for improving OSA diagnosis.

**Recent Findings:**

Based on a review of current literature, alternative metrics to the AHI such as the hypoxia burden, sleep apnea-specific pulse rate, and oxygen desaturation rate were shown to be correlated with indicators of CVD in OSA patients. A recent mathematical study also presents the possibility of a model-based metric to eliminate existing bias in diagnostics and to provide a more accurate quantification of tissue hypoxia.

**Summary:**

The analyzed studies give incentive to look beyond current clinical standards in OSA. Through this review, we motivate the use of mathematical modeling as a future avenue to improve OSA diagnosis with a hypoxia-based approach.

## Introduction

Obstructive sleep apnea (OSA), the most common respiratory disorder of sleep, is characterized by a repeated narrowing or collapse of the upper airway [1]. This leads to periods of reduced (hypopnea) or ceased (apnea) breathing, causing sleep fragmentation and cycles of oxygen desaturation and recovery [1]. The observed pattern of intermittent hypoxia (IH) in patients, a hallmark of OSA, is believed to be associated with several adverse health outcomes including cardiovascular disease (CVD) [2, 3]. In fact, the incidence of OSA in patients with heart failure, left ventricular dysfunction, hypertension, and coronary artery disease is as high as 40–80% [4]. Regardless of these alarming figures, OSA continues to be underdiagnosed and, consequently, undertreated in CVD patients [5]. In addition, it was estimated that OSA affects nearly 1 billion individuals aged 30–69 worldwide and that the prevalence in some countries is greater than 50% [6]. These concerning statistics are, in part, a result of the limitations in the diagnosis of OSA.

Currently, the standard diagnostic tool for OSA is polysomnography (PSG). A typical in-laboratory PSG involves overnight recording of nasal pressure or oronasal temperature to serve as surrogates for airflow pattern, abdominal and chest movement for respiratory effort, pulse oximetry for oxygen saturation and heart rate, electroencephalogram (EEG) signals to detect arousals and sleep stages, and electrocardiogram (ECG) signals for cardiac function [7]. The surrogate airflow or respiratory effort signals are then used to determine the apnea-hypopnea index (AHI), which is the primary clinical indictor of OSA severity. The AHI is calculated using predetermined criteria for scoring obstructive events [8] and often informs patient treatment decisions. However, the effectiveness of this clinical diagnostic standard has been questioned [9].

Along with concerns of clinical standard effectiveness, there are also disparities in the presentation and care of OSA. For example, a higher percentage of men are diagnosed with OSA than women [10], but it is not clear if this is due to less of a prevalence of OSA in women or a disparity in diagnosis. Indeed, women tend to report “atypical” symptoms of OSA compared to the standard [10, 11], which was likely set without sex-specific considerations [12]. Racial disparities also exist, with observed differences in the presentation and severity of sleep disturbances amongst specific groups [13]. Although further research is required to form concrete conclusions, some minorities are more prone to being diagnosed with cases of severe OSA [14], which is likely to affect the risk and development of comorbidities in patients. For example, African American patients were found to have higher adjusted odds of developing new-onset CVD in comparison with white patients [15]. Furthermore, recent works indicate the need to further assess social disparities in OSA [16, 17].

With clear challenges in OSA patient care, the purpose of this review is to provide a more detailed outlook on diagnosis and on how it is linked to OSA-related CVD in patients. To achieve this, we will analyze current limitations in primary clinical standards, assess any recent improvements, and suggest future avenues for further solutions.

### Association Between OSA and CVD and the Role of IH

IH is a unique pathophysiological feature of OSA, attributed to repeated respiratory obstructions during sleep. The recurrence of episodes of upper airway collapse, that are terminated by arousals throughout the sleep period, is the main abnormality of OSA. Each obstructive episode leads to a period of hypoxia followed by reoxygenation, initiated by an arousal and its associated hyperventilation. The recurrence of these fluctuations in oxygen leads to the characteristic IH pattern of OSA. Interestingly, the cycles of desaturation/reoxygenation are not sinusoidal, as desaturation time has been seen to have an approximate linear increase with desaturation severity, while reoxygenation time remains relatively constant [18]. Although the separate contributions of the hypoxia and reoxygenation phases towards CVD are not clear, the overall impact of IH has been extensively investigated.

Clinical research has demonstrated that OSA is an increased risk factor for hypertension [19, 20], which has serious cardiovascular consequences. Several studies have indicated a causative relationship between IH and vascular endothelial dysfunction (VED) (Table 1), which has a functional link to the development of hypertension [21, 22]. These investigations (Table 1) provide support for mechanisms of VED such as IH-induced vasoactive species imbalance, inflammation, and reactive oxygen species (ROS) production [21]. As an example, recent human studies evaluating biopsied endothelial tissue [23] and blood serum biomarkers [24] found an upregulation of endothelin 1 (ET-1), a vasoconstrictor and predictor of incident heart failure [25], in patients with OSA when compared to those without OSA. Similarly, IH exposure of human coronary artery endothelial cells caused an increase in ET-1 mRNA and protein expression, along with a decrease in nitric oxide (NO), a vasodilator, production [26]. Limited NO availability is likely a result of reduced endothelial nitric oxide synthase (eNOS) function, which can be caused by proteins such as Caveolin 1 (cav-1) or C-reactive protein (CRP). Indeed, IH-related increases in cav-1 and CRP, which is also a predictor of cardiovascular consequences [27], have been observed in in-vitro [26] and human [28] studies, respectively. Furthermore, inflammation induced by IH has also been observed. In one in-vitro study, culturing endothelial cells under IH and shear stress revealed the activation of pro-inflammatory pathways, with an up-regulation of intercellular adhesion molecule 1 (ICAM-1) and CC-chemokine ligand 2 (CCL2) when compared to the control group, along with increased expression of phosphorylated extracellular signal-regulated kinase (ERK) and IkB protein [29]. Moreover, interleukin 6 (IL-6), a pro-inflammatory cytokine, has been shown to be upregulated in OSA patients, with a statistically significant difference compared to the control group [30]. Other human studies have also demonstrated a significant impact of OSA on VED including overexpression of adhesion molecules [31] and inflammatory markers [32], activation of the renin-angiotensin system [33], and increase in vascular oxidative stress [34, 35].

**Table 1:** Recent studies elucidating the link between OSA and CVD

Subject	Intermittent Hypoxia Protocol	Results	Study
In-vitro Experiments			
Human coronary artery endothelial cells	Cycles of 30 min 0.1% O_2_ + 5% CO_2_ and 30 min 21% O_2_ + 5% CO_2_ repeated 9 times/day for 3 days (protein analysis) or 1 day (RNA analysis)	Increased protein and mRNA expression of ET-1 and cav-1 and decreased tonic NO generation (all *P* < 0.05 vs. control)	[26]
Primary human umbilical vein endothelial cells	Hypoxic medium (95% N_2_ + 5% CO_2_) perfused 5 or 15 times/hour for 4 hours (duration of desaturation and reoxygenation determined using patient PSGs)	Increased mRNA expression of ICAM-1 and CCL2 and increased protein expression of phosphorylated ERK and IkB (all *P* < 0.05 vs. control for 15 hypoxia events/hour condition)	[29]
Animal Studies			
Male Wistar rats	3-minute cycles (each cycle consisting of 5% O_2_ + 5% CO_2_ followed by an air flush) for 7 hours/day and 4 weeks	Increased right ventricular systolic pressure (*P* < 0.05 vs. control)	[36]
Male Sprague-Dawley rats	2-minute cycles (O_2_ decreased to 6% for 30 s, maintained for 50 s, and increased to 21% for 40 s) for 8 hours/day and 6 weeks	Ex-vivo analysis of cardiomyocyte injury with disorganization, necrosis, and edema (*P* < 0.05 for number of necrosis and edema cells vs. control) Increase in diastolic and systolic blood pressures, atrial dilation, ventricular hypertrophy, and decreased ejection fraction (all *P* < 0.05 vs. control)	[37]
Male Wistar rats	60 cycles/hour (30 s of 21% O_2_ and 30 s of 5% O_2_ for each cycle) for 8 consecutive hours/day and 14 days	Increase in systolic, diastolic, and mean arterial blood pressures, as well as increased ventricular fibrillation after inducing in-vivo and ex-vivo myocardial ischemia (all *P* < 0.05 vs. control)	[38]
Male Sprague-Dawley rats	Approximately 60 cycles/hour (Decrease O_2_ to 4–6%, hold at nadir for 3–5 s, and reoxygenate to 21% for each cycle) for 8 hours/day, 5 days/week, and 5 weeks	Increase in LV free wall weight to heart weight ratio, LV end-diastolic pressure, LV end-diastolic and end-systolic diameters and volumes, and LV oxidative stress Decrease in LV ejection fraction and LV percent fractional shortening (all *P* < 0.05 vs. control)	[39]
Human Studies			
Isolated micro-vessels from gluteal subcutaneous biopsies	-	Increased expression of ET-1, NOX-4, and ARG-1 in OSA patients (all *P* < 0.05 vs. control)	[23]
Isolated micro-vessels from gluteal subcutaneous biopsies	-	Increased expression of AT1 and AT2 in OSA patients (both *P* < 0.05 vs. control)	[33]
Isolated micro-vessels from gluteal subcutaneous biopsies	-	Decreased superoxide production in tissue from treated OSA patients (*P* < 0.0001 vs. pre-treatment) Increased superoxide production in tissue from OSA patients vs. non-OSA participants	[34]
Harvested venous endothelial cells with angiocatheter insertion into forearm	-	Increase in COX-2 and iNOS expression in cells from OSA patients (both *P* < 0.05 vs. control)	[32]
Venous blood sample	-	Increased superoxide release from OSA patient polymorphonuclear neutrophils after stimulation (*P* < 0.05 vs. controls)	[35]
Venous blood sample	-	Increase in ICAM-1 and VCAM-1 in OSA patients without treatment (*P* < 0.05 when comparing all treatment groups (full, partial, and none) for ICAM-1 (2-year follow up) and VCAM-1 (baseline))	[31]
Venous blood sample	-	Increase in CRP expression with OSA severity (*P* < 0.0001 for correlation with AHI)	[28]
Venous blood sample	-	Increase in ET-1 in OSA group (*P* < 0.001 vs. control group)	[24]
Venous blood sample	-	Increase in IL-6 in OSA group (*P* < 0.001 vs. control group)	[30]

*ARG-1* arginase, *AT1* angiotensin receptor type 1, *AT2* angiotensin receptor type 2, *cav-1* Caveolin 1, *CCL2* CC-chemokine ligand 2, *COX-2* cyclooxygenase-2, *CRP* C-reactive protein, *ERK* extracellular signal-regulated kinase, *ET-1* endothelin 1, *ICAM-1* intercellular adhesion molecule 1, *IL-6* interleukin 6, *iNOS* inducible NO synthase, *LV* left-ventricular, *NADPH* nicotinamide adenine dinucleotide phosphate, *NO* nitric oxide, *NOX-4* NADPH oxidase subunit 4, *PSG* polysomnography, *VCAM-1* vascular cell adhesion molecule 1

In addition to the evidence of molecular mechanisms of IH-induced VED and hypertension, animal models of OSA have also indicated a link between IH and CVD-related consequences (Table 1). In one study, chronic IH exposure in rats was found to cause cardiomyocyte injury, with visible disruptions in organization, necrosis, and edema [37]. Moreover, there was an indication of myocardial dysfunction with atrial dilation, left ventricular hypertrophy, and reduced ejection fraction [37]. Additional studies in rats have also observed IH-induced ventricular dysfunction with one showing elevated right ventricular systolic pressure in the IH group [36], which is indicative of pulmonary hypertension, and another showing increased ventricular fibrillation in the IH group after inducing regional myocardial ischemia in-vivo and ex-vivo [38]. Focusing on the left-ventricle, increases in end-diastolic pressure, end-diastolic and end-systolic diameters and volumes, and oxidative stress have been induced by chronic IH in rats, along with decreases in ejection fraction and percent fractional shortening [39]. Furthermore, chronic IH has also been shown to cause a significant increase in diastolic, systolic, and mean arterial blood pressures in rats when compared to the control group [38].

Regardless of this extensive evidence suggesting a direct causative link between IH during OSA and CVD, it is important to highlight that some studies have indicated a more complicated relationship. Perplexingly, a possible cardioprotective effect of IH has been hypothesized [40]. For example, in patients admitted for acute coronary syndrome, those with comorbid OSA were shown to have lower peak cardiac troponin 1 levels, a clinical marker of cardiac injury, when compared to those without OSA [41]. A separate study showed similar results with troponin-T levels decreasing with increasing OSA severity in patients admitted for acute, non-fatal myocardial infarction, suggesting an IH-induced preconditioning response [42]. In addition to this hypothesized cardioprotection of OSA, the lack of consensus on the effectiveness of continuous positive airway pressure (CPAP), the primary OSA treatment, has further alluded to more complexities in the link between OSA and CVD. If CVD was a direct result of IH, alleviating the breathing disturbances causing IH should, theoretically, improve the cardiovascular health of OSA patients. Yet, some clinical studies have not shown a prevention of cardiovascular events with CPAP [43]. Therefore, further research on the body’s intricate response to IH and its subsequent impact on the cardiovascular system will be essential for the field of sleep medicine.

### Limitations in Current Clinical Standards of Diagnosis

Similar to the unclear link between IH and CVD, current clinical standards have not indicated an evident relationship between OSA severity and CVD risk, which is interesting given that a high prevalence of CVD in patients with OSA has been observed. Recent large-scale observational studies have shown that the AHI is not a significant predictor of major adverse cardiovascular events (MACE) [44] or cardiovascular-related mortality [2] in all patient cohorts after adjusting statistical models for covariates such as age, sex, body mass index (BMI), smoking status, etc. Furthermore, there does not appear to be a strong association between the AHI and potential biomarkers of OSA severity in relation to CVD, such as ET-1 [24]. These results are interesting and raise the question of whether the observed poor correlations are a consequence of the effectiveness of current clinical standards.

The AHI, which is the primary clinical measure of OSA severity, has been identified as having limitations over the years. To calculate the AHI, obstructive events are scored as apneas or hypopneas based on the following American Academy of Sleep Medicine (AASM) criteria for adults, with several variations in literature: >=90% reduction in airflow lasting a minimum of 10-seconds for apneas and a >= 30% reduction in airflow lasting a minimum of 10-seconds, followed by a >= 3% oxygen desaturation or arousal for hypopneas [8]. In our recent work [45], OSA simulations were used to highlight the potential of misdiagnosis in patients due to the inclusion of a desaturation criteria for hypopneas and the inability of the AHI to account for event duration. Furthermore, we point out that the AHI does not reflect the temporal proximity of obstructive events or the severity of airflow reduction during hypopneas [45]. Another limitation is that the AHI relies on pulse oximetry for scoring hypopneas. This is problematic as pulse oximeters, in general, have been known to be affected by factors such as skin tone bias [46, 47]. Indeed, African American patients have been shown to have higher AHI scores than their white counterparts within specific age ranges, even after controlling for covariates such as BMI [48]. Along with these possible racial disparities, there are also sex disparities in OSA diagnosis. A recent study showed that using a 4% desaturation criteria for events resulted in a larger AHI difference between men and women, as compared to using a 3% criteria [49]. Sex differences also increased when excluding arousals in event scoring [49]. In addition, women have been shown to experience more severe desaturations following longer apneas when compared to men [50]. The higher prevalence of hypopneas observed in women compared to men for some OSA categories [51] and the fact that the AHI does not weight apneas differently based on the desaturation level could potentially lead to underdiagnosis of OSA or its severity in women. The discussed limitations in the AHI could be detrimental for patient treatment qualifications [52], potentially increasing exposure to unmitigated CVD risk.

### Potential Alternatives to the AHI

**Fig. 1:**
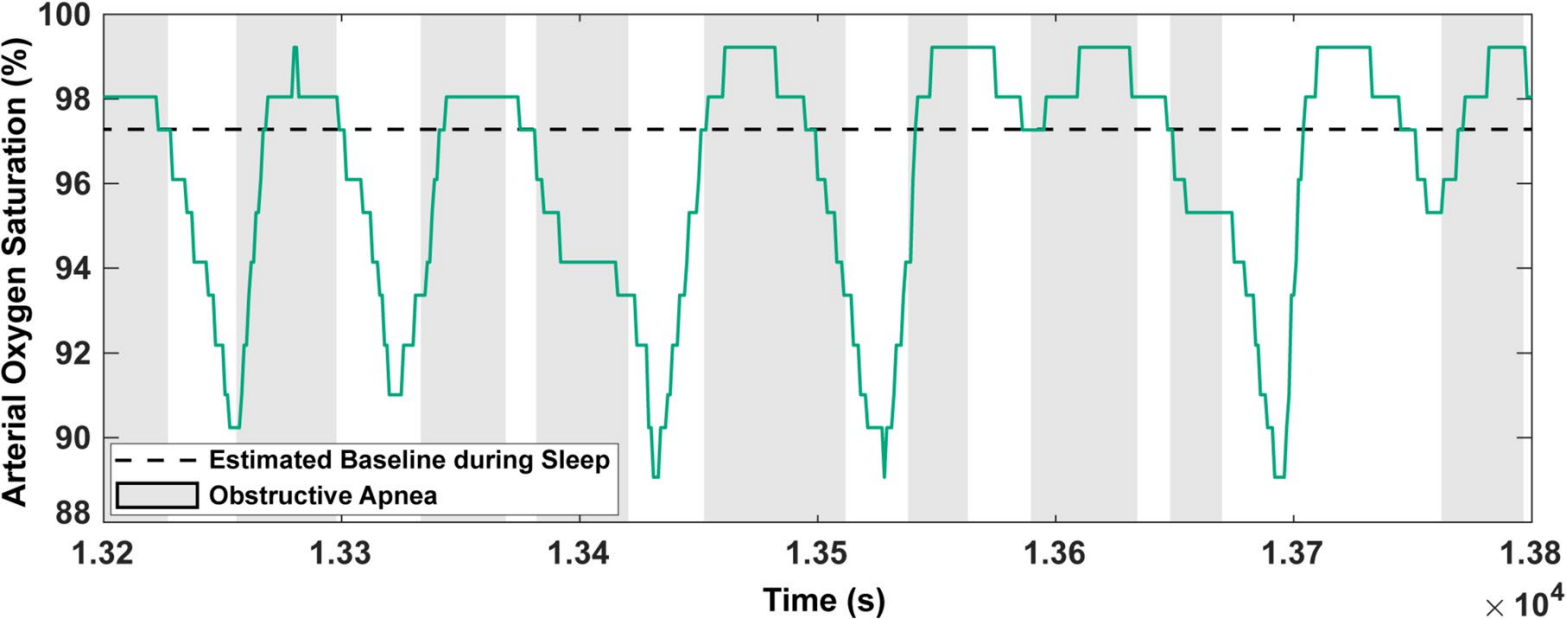
Pulse oximetry recording of OSA patient from the Sleep Heart Health Study. Dashed line represents an estimated baseline saturation for the patient recorded during normal sleep, without any breathing disturbances. Shaded gray areas represent periods of apnea

The discussion of diagnostic limitations above leads us to reviewing recent and possible future improvements in novel approaches to estimate disease severity in OSA. Considering the AHI, are there better predictors of OSA severity, specifically in association with comorbid CVD? Given the role of IH in the CVD risk and consequences of OSA, an emerging metric in literature has been the “hypoxia burden” (Table 2). An initial estimate of this quantity using the total time spent below 90% saturation (T90) was promising in its ability to predict mortality in heart failure with reduced ejection fraction (HF-REF) patients, unlike the AHI which was statistically insignificant in adjusted models [53]. However, one limitation of the T90 is that it does not separately consider OSA-related IH and hypoxia caused by other diseases or factors. Therefore, a more recent estimate of hypoxia burden has been defined using the area under the desaturation curve only associated with respiratory events [2]. This was also found to better represent OSA severity in respect to associated CVD consequences, specifically cardiovascular-related mortality [2] and incident heart failure in men [3], when compared to the AHI. Interestingly, the correlation between the mentioned measure of hypoxia burden and incident heart failure was not significant in women [3]. We identify two primary concerns with this approach to estimating hypoxia burden. First is its full reliance on pulse oximetry, also a problem with the T90. Pulse oximeter readings may be affected by skin pigmentation, which introduces the possibility of racial bias [46]. In fact, the two large-scale studies [2, 3] were predominantly done with white patients (> 80%). Additionally, pulse oximeters may suffer from movement artifacts, which can be quite prominent in highly disturbed sleep, as well as vasoconstriction and other issues [54]. Furthermore, a recent animal study suggests that pulse oximetry, although a valuable tool, may not provide a consistently accurate representation of deep-tissue oxygenation [55]. These factors will, therefore, affect all oximetry-derived metrics of hypoxia. Our second concern is the use of a pre-event baseline, the maximum oxygen saturation within a 100-second window prior to the end of an event [2], to calculate the desaturation area. Due to the occurrence of hyperventilation after events, the possibility of using a higher-than-normal oxygen saturation as a baseline may lead to an inaccurate representation of hypoxia burden (Fig. 1). One alternative is to choose a single baseline value, averaged across periods of normal, undisturbed sleep for each patient. There is also the possibility of using a physiologic baseline, the oxygen concentration at which cells begin to respond to hypoxia with occurrences such as hypoxia-inducible factor 1-alpha expression. Yet, this would require in-vitro experiments for baseline approximation as well as an estimation of tissue oxygenation during each sleep study. Other potential metrics for OSA severity are the sleep apnea-specific pulse rate and the oxygen desaturation rate (Table 2). The sleep apnea-specific pulse rate was defined using the difference between the maximum heart rate over a search window for each event and the event-associated minimum [56]. This metric was seen to be associated with increased cardiovascular risk [56]. The oxygen desaturation rate was defined using the ratio of saturation decrease (start of decrease to nadir) associated with an apnea and the desaturation length [57]. To ensure calculation only for instances of a closed system, hypopneas were not considered owing to the possibility of oxygenation during partial airflow [57]. The oxygen desaturation rate was seen to be correlated with wake systolic blood pressure, asleep systolic blood pressure, and short-term blood pressure variability in patients with OSA (some having comorbid hypertension) [57]. Although these appear to be promising metrics, they do not provide a quantification of IH at the tissue-level. We believe that mathematical modeling can improve PSG-based risk classification by providing tissue oxygen levels as an additional data stream to pulse oximetry.

**Table 2:** Current and proposed indicators of OSA severity

Indicator	Definition	Source for calculation	Effectiveness in OSA Patients with Comorbid CVD	Status of Use
AHI	Number of apneas and hypopneas per hour of sleep	Predetermined criteria [8] and pulse oximetry	Not significantly correlated with/predictor of cardiovascular-related mortality [2], incident cardiovascular events [44], heart failure [3], and systolic blood pressure [57] after adjusting for covariates	Clinical standard
T90	Total time spent below 90% saturation	Pulse oximetry	Associated with MACE [44] and mortality in stable HF-REF patients [53]	Proposed in literature
Hypoxia Burden	Total area under desaturation curve over search windows associated with respiratory events, normalized by sleep duration. Respiratory events identified using only amplitude reduction [2]	Pulse oximetry	Associated with, MACE [44], cardiovascular-related mortality [2] and heart failure in men [3]	Proposed in literature
Sleep Apnea-Specific Pulse Rate	Mean of all event-related responses, with each response being a difference between the maximum heart rate over an event-specific search window and the event-associated minimum [56]	Pulse oximetry	Associated with increased cardiovascular risk [56]	Proposed in literature
Oxygen Saturation Rate	Rate of oxygen desaturation associated with apneas [57]. Each rate calculated as a ratio of the extent of desaturation (start to nadir) and desaturation time	Pulse oximetry	Correlated with systolic blood pressure and short-term blood pressure variability [57]	Proposed in literature
Hypoxia Burden Score	Area deviation of dissolved oxygen concentration from baseline (average value over wake stage), normalized by total time [45]	Mathematical model	Not tested	Proposed in literature

### A Call for Action: Hypoxia-Based Modeling of OSA Severity

**Fig. 2:**
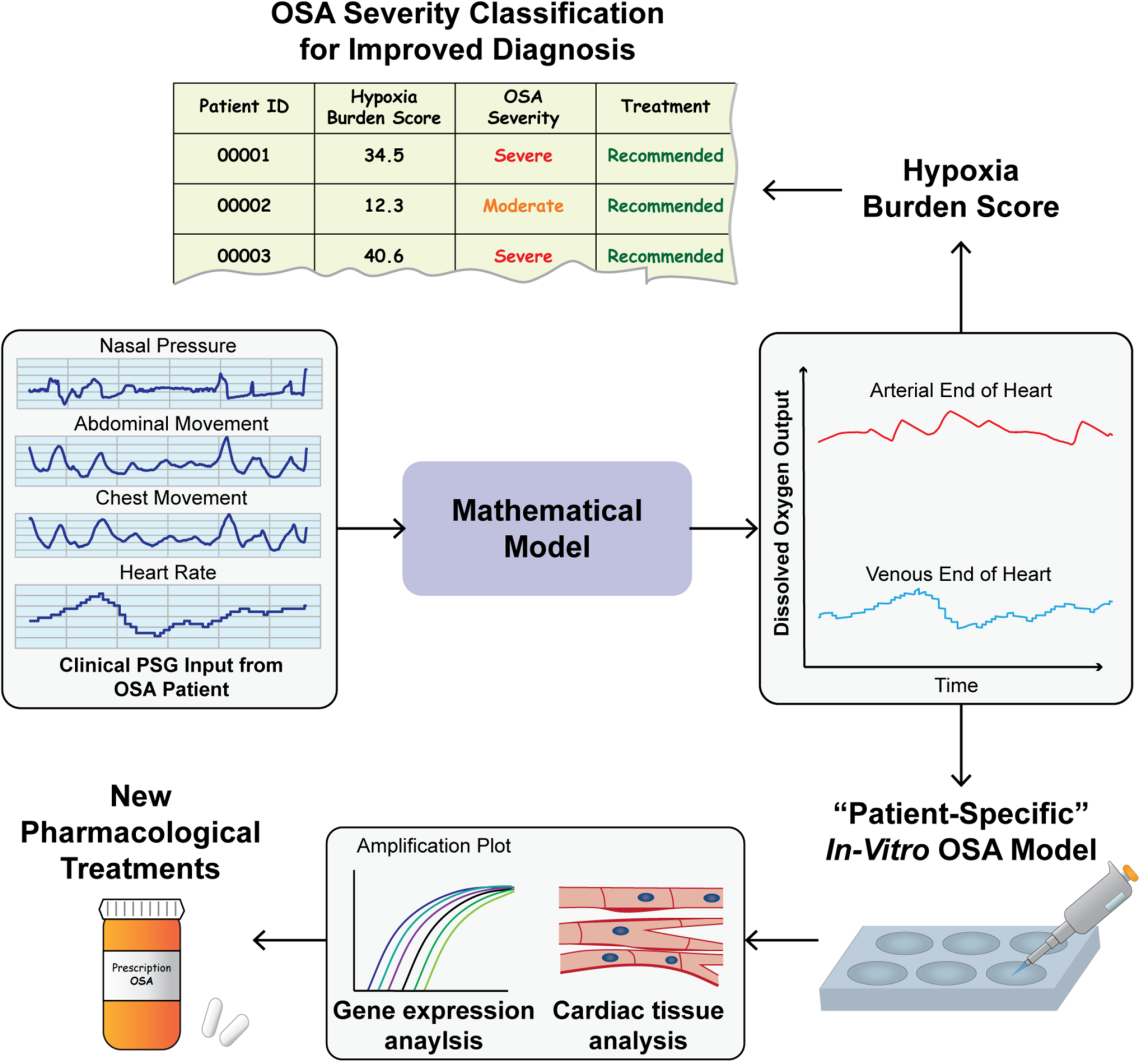
Application of mathematical modeling towards OSA patient care

The ideal estimate of IH exposure in patients with OSA should rely on reproducible clinical signals obtained from sleep studies but also account for the dynamic physiological and pathophysiological responses to respiratory events. Therefore, a mathematical model accounting for the cardiorespiratory response to obstructions can provide an accurate estimation of tissue hypoxia exposure during each respiratory event, with the ability to further stratify the severity of each event (a function not available in the current AHI) and to generate an overall index of clinically significant events that result in tissue hypoxia. Although there have not been many recent mathematical models of OSA, we believe that this approach can be used to improve the understanding of OSA and CVD and to improve patient care (Fig. 2). The ideal model would be one capable of direct clinical application, by incorporating patient data from PSG as an input (Fig. 2). Previous studies have attempted to simulate the impact of OSA by developing an integrative model of the cardiovascular, respiratory, and sleep regulation systems [58], along with metabolic effects [59]. However, these models are increasingly complex, with hundreds of required parameters for simulation, and do not incorporate patient-specific PSG inputs, which severely limits their applicability to clinical care. In addition, there is not a spatial model of gas transport in the pulmonary capillaries, which may become essential in cases of severe OSA [45]. To overcome these concerns, and maximize clinical relevance, we recently developed a mathematical model which uses patient-specific nasal pressure and heart rate from PSGs as an input and outputs dissolved oxygen concentration [45]. The model formulation was based on fundamental mass transfer principles, focusing on oxygen transport between the external environment and alveoli, the alveoli and pulmonary capillaries, and the systemic capillaries and body tissues [45]. Using the dissolved oxygen output, we created a new definition of hypoxia burden as the normalized area deviation from a set baseline [45]. Although this proposed score has not yet been tested in large datasets of OSA patients with comorbid CVD and can be further optimized, it makes use of the dissolved oxygen concentration, which is essential to consider for IH as this is what the tissues are directly exposed to. Moreover, with this model-based score approach, we are able to avoid the issues associated with pulse oximetry. To further improve specificity towards CVD, our model can be adjusted to recalculate the hypoxia burden score for a heart capillary compartment (Fig. 2).

Yet, to fully utilize the potential of this approach, there is an existing gap between current clinical information and ideal model inputs that must be addressed. Currently lung volumes are not recorded in typical sleep studies, which tend to rely on a qualitative assessment of surrogate airflow or respiratory signals to detect obstructions, rather than providing an absolute quantification of breathing. However, our model makes use of cumulative alveolar volume as a time-varying breathing input [45]. As a result, to allow for clinical application, one of the PSG signals must be converted to alveolar volume. Unfortunately, this approach has not been adequately studied. Previous works have obtained time-varying volume estimates by using recorded nasal pressure signals [45, 60]. However, this method may become erroneous during respiratory events with negative pressure swings [45] or during oral breathing. Therefore, it will be necessary to obtain an accurate estimate of alveolar volume by optimizing the conversion process, perhaps by using a combination of PSG signals. In addition to the aforementioned hurdles towards clinical application, it is important to address the general limitations of mathematical modeling. Despite the usefulness of models, they cannot fully replicate the body’s natural responses. Therefore, it is imperative to identify the essential components of the target system to ensure that the model realistically represents it, while still allowing for feasible clinical application. Regarding OSA, spatial mass transfer along the lungs and patient-specific inputs are important elements which have not been adequately represented in all previous models of the disease.

### Application of Hypoxia-Based Models Towards “Patient-Specific” Experiments

One advantage of a hypoxia-based model approach is that it can also be applied towards improving experimental models of OSA. Previous in-vitro studies have mainly used generic patterns of IH to simulate OSA [61, 62], which do not adequately capture in-vivo behavior. Taking a different direction, a recent work analyzed severe OSA patient PSGs to produce the IH pattern imposed on cultured endothelial cells [29]. Although this study presents a promising approach for a more pathophysiologically accurate in-vitro model, it focuses only on severe desaturation cycles (SpO_2_ < 80%) from PSG data [29]. A more relevant model would be one that captures the total impact of all events during a sleep study, without relying on pulse oximetry data. Therefore, in our opinion, a model-based hypoxia burden can be used to create “patient-specific” IH protocols for experimental models of OSA to better understand impact on gene expression, cardiac tissue structure and function, and to even test new pharmacological treatments. Mass transfer in a well plate can be modeled using a fundamental equation, and, with continuous monitoring of oxygen levels in a hypoxia chamber, the hypoxia burden on cultured cells can be calculated similarly as in previous works [45]. The IH protocol can then be adjusted until the score for cultured cells is the same as that for a specific patient. We believe that this approach will bridge the gap between clinical data and in-vitro representations for a better understanding of the effect of OSA on cardiac health.

## Conclusions

In this review, we analyzed the current standard of care for the diagnosis of OSA. Specifically in relation to CVD, recent papers have highlighted the ineffectiveness of the AHI, which points to the need for improved measures. The idea of using a burden of hypoxia to quantify OSA severity is promising, and we propose to apply mathematical modeling to further optimize its definition. A mathematical approach will not only help to eliminate existing disparities in diagnostics, but it will also better inform “patient-specific” experimental models of OSA for better understanding the association with CVD. Although further optimization of mathematical models is necessary, this approach introduces an exciting avenue for improving OSA patient care.

## Key References

- Gavrilin MA, Porter K, Samouilov A, Khayat RN. Pathways of Microcirculatory Endothelial Dysfunction in Obstructive Sleep Apnea: A Comprehensive Ex Vivo Evaluation in Human Tissue. Am J Hypertens. 2022;35:347–55.
  - This study highlighted possible endothelial dysfunction in OSA patients, with elevated endothelin-1 observed in an ex-vivo analysis.
- Trzepizur W, Blanchard M, Ganem T, Balusson F, Feuilloy M, Girault J-M, et al. Sleep Apnea–Specific Hypoxic Burden, Symptom Subtypes, and Risk of Cardiovascular Events and All-Cause Mortality. Am J Respir Crit Care Med. 2022;205:108–17.
  - The authors show that hypoxia burden is correlated with MACE in OSA patients and performs better than the AHI.
- Qayyum NT, Wallace CH, Khayat RN, Grosberg A. A mathematical model to serve as a clinical tool for assessing obstructive sleep apnea severity. Front Physiol. 2023;14.
  - This study demonstrates the use of mathematical modeling, with simulated inputs to identify limitations in the AHI and patient-specific PSG inputs to calculate hypoxia burden scores.
- Azarbarzin A, Sands SA, Younes M, Taranto-Montemurro L, Sofer T, Vena D, et al. The Sleep Apnea–Specific Pulse-Rate Response Predicts Cardiovascular Morbidity and Mortality. Am J Respir Crit Care Med. 2021;203:1546–55.
  - This study presents the sleep apnea-specific pulse rate as a potential indicator of increased cardiovascular risk in OSA patients.

## Data Availability

No datasets were generated or analysed during the current study.

## Declarations

The Section Editor for the topical collection Sleep and Cardiovascular Disease is Rami Khayat. Please note that Section Editor Rami Khayat was not involved in the editorial process of this article as he is a co-author.
